# Green Onion (*Allium fistulosum*): An Aromatic Vegetable Crop Esteemed for Food, Nutritional and Therapeutic Significance

**DOI:** 10.3390/foods12244503

**Published:** 2023-12-16

**Authors:** Seong-Hoon Kim, Jung Beom Yoon, Jiwon Han, Yum Am Seo, Byeong-Hee Kang, Jaesu Lee, Kingsley Ochar

**Affiliations:** 1 National Agrobiodiversity Center, National Institute of Agricultural Sciences, RDA, Jeonju 5487, Republic of Korea; 2 National Institute of Horticultural and Herbal Science, RDA, Wanju 55365, Republic of Korea; beomi7944@korea.kr; 3 National Institute of Horticultural and Herbal Science, RDA, Muan 58545, Republic of Korea; support06@korea.kr; 4 Department of Data Science, Jeju National University, Jeju 63243, Republic of Korea; seoya@jejunu.ac.kr; 5 Department of Applied Plant Science, Chonnam National University, Gwangju 61186, Republic of Korea; rkdqudgml555@naver.com; 6 Korea Partnership for Innovation of Agriculture, RDA, Jeonju 54875, Republic of Korea; butiman@korea.kr; 7 Council for Scientific and Industrial Research, Plant Genetic Resources Research Institute, Bunso P.O. Box 7, Ghana

**Keywords:** *Allium fistulosum*, bioactive compound, flavor, green onions, nutrition, phytochemical

## Abstract

In recent years, there has been a shift towards a greater demand for more nutritious and healthier foods, emphasizing the role of diets in human well-being. Edible *Alliums*, including common onions, garlic, chives and green onions, are staples in diverse cuisines worldwide and are valued specifically for their culinary versatility, distinct flavors and nutritional and medicinal properties. Green onions are widely cultivated and traded as a spicy vegetable. The mild, onion-like flavor makes the crop a pleasant addition to various dishes, serving as a staple ingredient in many world cuisines, particularly in Eastern Asian countries such as China, Japan and the Republic of Korea. The green pseudostems, leaves and non-developed bulbs of green onions are utilized in salads, stir-fries, garnishes and a myriad of culinary preparations. Additionally, green onions have a rich historical background in traditional medicine and diets, capturing the attention of chefs and the general public. The status of the crop as an important food, its culinary diversity and its nutraceutical and therapeutic value make it a subject of great interest in research. Therefore, the present review has examined the distribution, culinary, nutritional and therapeutic significance of green onions, highlighting the health benefits derived from the consumption of diets with this aromatic vegetable crop as a constituent.

## 1. Introduction

**Figure 1:**
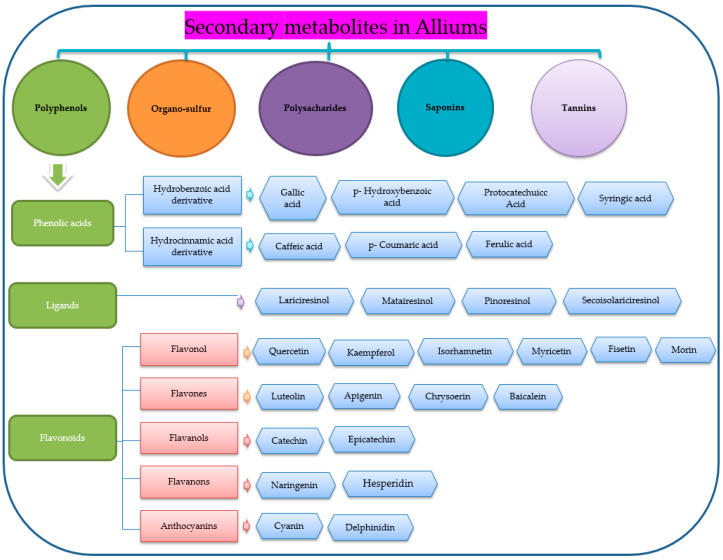
Current state of research on secondary metabolites in *Alliums*.

In recent years, significant changes on a global scale have been observed, with a noticeable shift towards the increased demand and consumption of healthier and nutritious foods, reflecting a growing awareness of the importance of diets in contributing to the overall human well-being [1,2,3]. This transformation of eating habits arises, in part, from the increasing knowledge about the association between certain food consumption and rates of disease occurrences and control [4,5]. This knowledge has accounted for the reason behind the surge in the consumption of vegetable crops known to contain different kinds of health-promoting substances, such as vegetables [3,6]. Vegetable crops are rich in vitamins, minerals, antioxidants and dietary fiber and thus contribute to better nutrition and prevent or help manage many human health disorders [5,7,8]. As individuals and societies prioritize health-conscious choices, the consumption of such nutrient-rich vegetables will continue to grow, fostering a positive shift toward healthier eating habits [2]. The plant genus *Allium* contains a variety of vegetable species famous for their pungent, spicy properties, usually consumed raw in salads or in cooked forms [9]. The cultivation of *Allium* vegetables contributes to global food and nutritional security by providing a reliable source of sustenance year-round [10]. *Allium* species produce a diverse array of secondary metabolites, encompassing polyphenols, organosulfur compounds, saponins, polysaccharides and tannins [11] (Figure 1). These compounds are not only responsible for the characteristic flavors and aromas associated with *Allium* species but they are also essential in preventing several human diseases [12]. For instance, the bioactive constituents of these crops form the basis for their actions as antimicrobial, antioxidant, antitumor, immunoregulatory, antidiabetic, antiobesity and anti-inflammatory properties, thus showcasing their therapeutic value in maintaining and promoting human health, especially when incorporated into varied forms of diets [13]. In fact, the versatility of edible Alliums in both culinary and medicinal applications has consolidated their status as essential ingredients in kitchens worldwide [14].

Green onions (*Allium fistulosum*), also known as scallion, Japanese bunching onion, Welsh onion or spring onion, are an important member of the Amaryllidaceae family [12], characterized by their distinct flavors and culinary versatilities [15]. *A. fistulosum* is widely cultivated and traded as a spicy vegetable [16]. Its genetic makeup is characterized by two sets of chromosomes (diploid species) with 16 chromosomes (2n = 2X = 16) [17]. The wide-spread nature of green onions and its recognition in the global spicy vegetable markets reflect the valuable contribution of the crop to global food security, culinary diversity and nutraceutical benefits [18]. The crop is endowed with a mild and onion-like flavor, which is a valuable addition to a wide range of dishes and as a staple ingredient in many cuisines [19]. It is particularly valued for its green pseudostems, leaves and non-developed bulbs, used in salads, stir-fries, garnishes and various culinary preparations [14,20]. The crop has been an integral component of human diets and traditional medicine for centuries, making it a subject of great interest to researchers, health practitioners, chefs and the general public [21]. In particular, green onions hold significant culinary importance in Eastern Asian countries such as China, Japan and the Republic of Korea [22]. In Western countries, green onion is primarily consumed as a scallion or salad and is the most commonly marketed species for this purpose [7]. This review, focusing on *A. fistulosum*, is motivated by the growing recognition of the crop as a significant vegetable cultivated and traded worldwide for its distinct flavor and culinary versatility. The status of the crop as an important food security, culinary diversity and nutraceutical resource makes it a subject of great interest in research. Therefore, the present review examined the distribution, culinary, nutritional and therapeutic significance of the aromatic vegetable crop *Allium fistulosum*.

## 2. Characteristics of *Allium fistulosum*

**Figure 2:**
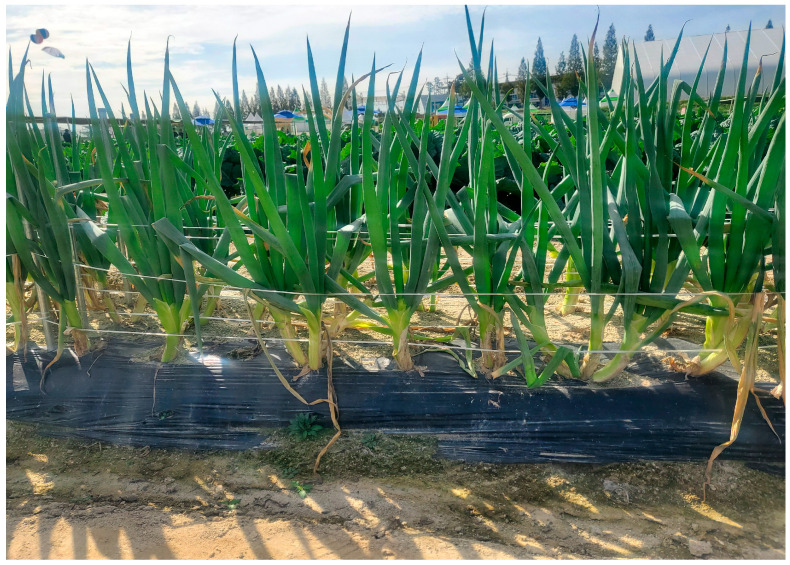
*Allium fistulosum* plants grown in the experimental field at the Agrobiodiversity Center, RDA, the Republic of Korea.

Green onion, a perennial herbaceous vegetable crop, exhibits distinct agronomic and morphological variations that are of interest to botanists, horticulturists and culinary enthusiasts alike [23]. Taxonomically, green onion belongs to the division angiosperms, class Liliopsida, order Asparagale, family Amaryllidaceae, genus *Allium* and species *fistulosum*. The crop has unique botanical features that make it distinct from its close relatives, the common onions (*Allium cepa*), leeks (*Allium ampeloprasum*), chives (*Allium schoenoprasum*) and garlic (*A. sativum*) [24]. For instance, though they have a mild onion-like flavor, unlike the bulb-forming *Allium* species (such as common onions and garlic), green onions are generally non-bulb-producing and notable for their slender green stalks [25] used in traditional medicine and mild onion flavor [26]. The leaves of *A*. *fistulosum* are tubular or hollow (*fistula*) in nature and can grow up to 60 cm in length [26]. It typically displays more upright or erect leaves, setting it apart from *A. cepa*, which often has more sprawling foliage [22]. Figure 2 shows green onions from the Rural Development Administration (RDA) Genebank, grown in the field located at the National Agrobiodiversity Center, the Republic of Korea, which reveals their slender, hollow green leaves. They produce spherical, umbrella-like inflorescences composed of small, star-shaped white or pale purple bisexual flowers [14].

From a taxonomist perspective, *A. fistulosum* cultivars and varieties show variations in terms of stem color (green, white, red, or purple), stem thickness, leaf length (color, size, shape, length), bulb formation and flavor intensity, contributing to the overall diversity of the species [27]. For agronomic traits, uniformity, disease resistance, plant growth habit and earliness are important considerations for growers and breeders [14]. The characteristically stronger pungency of *A. fistulosum* contributes to the crop’s unique flavor profile [28]. Also, among green onions, a considerable amount of morphological diversity exists [28]. These characteristics are essential to farmers, horticulturists and culinary experts in selecting the most suitable varieties for specific purposes, such as garnishes, ingredients in salads and as essential components in various dishes around the world [14]. Alliums are also generally hardy and adaptable to diverse environmental conditions, making the crops easily cultivated by home gardeners and commercial growers, irrespective of the field of protected conditions [20]. The adaptable nature of the crop makes it useful for consumption throughout the year, bridging seasonal gaps in fresh produce availability.

## 3. Countrywide Distribution of Green Onions

**Figure 3:**
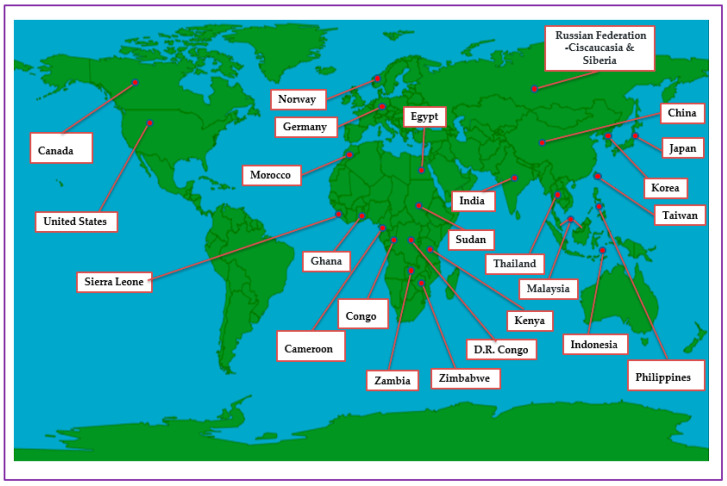
Global distribution of green onions.

In terms of distribution, though green onions are cultivated in many areas across the globe (Figure 3), they are highly popular in East Asia, ranging from Siberia to tropical Asian countries such as China, Vietnam, Taiwan, Japan, the Philippines, the Republic of Korea, Malaysia and Indonesia [22]. China is the world’s topmost green-onion-producing country, with an estimated production area that exceeds 500,000 ha, ahead of production areas in Japan and the Republic of Korea, each of the latter two countries with an approximate production area of 25,000 ha [14]. Significant production of green onions also occurs in America, Europe and Africa [10,20]. In Europe, Germany has the biggest green onion production area, estimated at 1300–1400 hectares [14]. For Africa, green onion production, mainly for domestic consumption, occurs in countries including Ghana, Sudan, Kenya, Cameroon, Congo, D.R. Congo, Sierra Leone, Zambia and Zimbabwe [20]. Yet, in Africa, larger production occurs in Egypt and Morocco (Egypt: ~4000 ha, and Morocco: ~350–400 ha), which are key suppliers of fresh green onions in European markets [29]. Green onions thrive in temperate, subtropical and tropical conditions [20]. The crop’s adaptability to different climates and growing conditions reveals its widespread cultivation and consumption nature [30]. The crop’s growth preferences include well-drained, fertile soils and a variety of soil types, from sandy to loamy [31]. While it is relatively drought-tolerant, consistent moisture is essential for the optimal growth of the crop. Adequate watering is crucial, especially during dry spells [32]. This adaptability to diverse conditions is positive, being the basis for the year-round cultivation and consumption of the crop in different regions. This further ensures a steady supply of fresh green onions in local markets, contributing to their global popularity [23].

## 4. Cultural and Culinary Applications

Green onions have been cultivated and consumed for centuries across various cultures [33]. At present, the cultivation and consumption of the crop have transcended geographical boundaries, becoming an integral part of many culinary traditions [24]. Different cultivars of green onions exhibit variations in appearance and pungent flavor and aroma, influenced mainly by their organosulfur compounds [34]. The taste can vary from mild and sweet to pungent and spicy [35]. Different culinary traditions rely on green onions as a versatile ingredient, and the choice of variety can significantly affect the taste and aroma of a dish [36]. Milder green onions are often favored for their subtle onion flavor, making them a delightful addition to salads, garnishes and dishes where a gentler onion taste is desired. On the other hand, the spicier varieties impart a more robust and pungent onion flavor, which adds depth and character to stir-fries, soups and savory dishes [37]. Green onions are popularly used as ingredients in various Asian and Latin American cuisines [7,38]. They can be eaten in both raw forms and in cooked dishes, adding a fresh crunch and mild onion flavor [14]. The white parts of green onions are relatively sharper, while the green tops are milder, creating a balance of tastes that can range from gentle to subtly tangy. This rich variation caters for the diverse and evolving culinary preferences of people, making green onions a versatile ingredient in various cuisines [39]. This range of flavors and textures allows chefs and culinary professionals to exercise their creativity and expertise, using different green onion varieties to craft unique and innovative culinary experiences. Chefs appreciate green onions not only for their distinct flavors but also for their contrasting textures. The crisp, succulent white and light green portions provide a satisfying crunch, while the tender green tops offer a refreshing and vibrant contrast. These textural variations make green onions not just a flavor enhancer but also a versatile ingredient for both visual and sensory appeal in dishes.

## 5. Nutritional Significance of Green Onions

Nutrients are essential for the survival and proper functioning of all living organisms. These chemical compounds, mainly vitamins and minerals, serve as the building blocks and catalysts for various physiological processes within the body [40]. To ensure that the body receives an adequate supply of these nutrients, maintaining a well-balanced diet is crucial. Balanced diets, encompassing a range of food groups, offer a unique set of nutrients necessary for optimal health and bodily function [41]. Green onions are an important source of many essential nutrients, offering a rich array of vitamins, minerals, dietary fiber, proteins, carbohydrates and phytochemicals in various culinary dishes [42]. Whereas macronutrients, encompassing proteins, carbohydrates and fats, represent the foremost sources of energy in the human body and are involved in various metabolism processes, micronutrients (minerals and vitamins) play critical cellular functions, serving as important antioxidants and participating in diverse enzymatic reactions [40]. These nutrients contribute to the overall well-being of individuals, aiding in the maintenance of proper bodily functions and the prevention of nutritional deficiencies.

### 5.1. Vitamins

**Figure 4:**
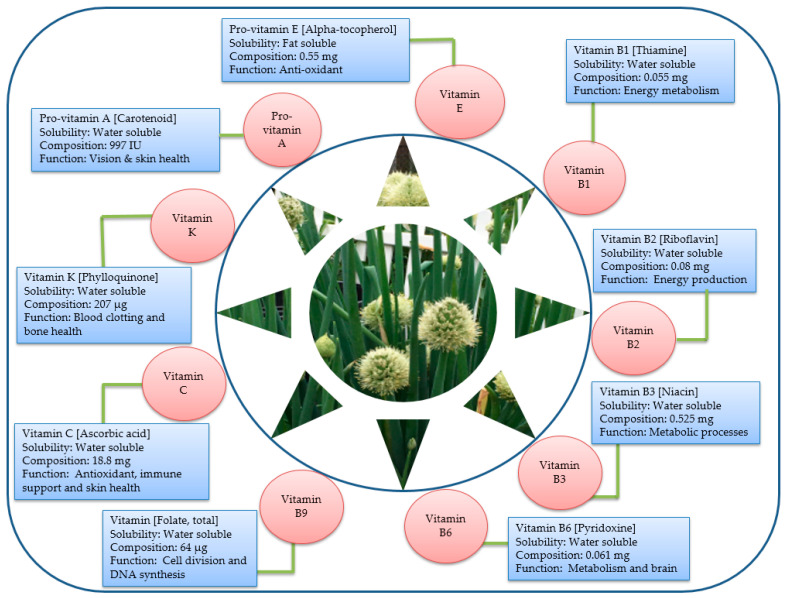
Solubility, nutritional composition and biological function of key vitamins in *A. fistulosum*.

Vitamins are indispensable for building a healthy human body, but the human body cannot synthesize them [43]. So, the consumption of diets rich in vitamins is the main source of the body’s vitamin requirement [44]. Despite being required in smaller quantities in the body [40], vitamins are pivotal in various metabolic pathways in living organisms, contributing to essential physiological functions [45]. Green onions contain a range of vitamins that are essential for various metabolic processes in the body [46] (Figure 4). More importantly, most of these vitamins are associated with important biological activities; for instance, they serve as antioxidants and anticancer, antimicrobial and antiobesity agents [7]. The major vitamins reported in green onions include vitamin A (in the form of pro-vitamin A-beta carotene), a range of vitamin B derivatives, including thiamine (B1), riboflavin (B2), niacin (B3), pyridoxine (B6) and folate (B9), and vitamin C [47]. These vitamins are vital for energy metabolism, nervous system function, DNA synthesis and overall human well-being [45].

Vitamin A in green onions is primarily present in the form of beta-carotene, a precursor to active vitamin A [48], which, as a component of the light-absorbing molecule in the eye’s retina, plays a critical role in maintaining healthy vision [45]. Vitamin A is also essential for cellular differentiation, gene expression, growth, healthy skin, healthy immune system function, bone development and reproduction [49]. Thiamine aids in converting food into energy and plays an essential role in maintaining a healthy nervous system [50]; riboflavin supports tissue growth [51]; niacin is crucial for skin health, metabolic processes, the central nervous system and energy production [52]; pyridoxine plays a role in brain development and the synthesis of neurotransmitters [50]; and folate is essential during pregnancy for fetal development.

The diverse array of B vitamins in green onions underscores their nutritional value and their potential to contribute to overall health and vitality. A comparative analysis indicates that the leaves of green onions contain higher amounts of vitamins B1, B2, B3 and C, carotenoids and minerals relative to the pseudostem [53]. In a study that investigated the nutritional composition and antiobesity effects of mixed *Allium fistulosum* and *Viola mandshurica* extracts (AFE + VME) in high-fat-diet-induced obese mice, Sung et al. [47] detected the presence of higher amounts of several nutrient elements, including vitamin B (B1, B2, B3 and B9). The presence of these nutritional elements, along with various bioactive compounds, was found to control obesity and various metabolic disorders.

Vitamin C, also known as ascorbic acid or ascorbate, is a crucial water-soluble vitamin that plays a multifaceted role in the body. It acts as a reducing agent in various enzymatic reactions, helping to facilitate critical physiological processes [54]. Additionally, vitamin C serves as a soluble antioxidant, protecting cells and tissues from damage caused by free radicals [55]. While vitamin C is found primarily in fruits and vegetables, it is interesting to note that animal organs like the liver and kidneys also contain this vitamin, highlighting its importance in both plant- and animal-based diets and its essential role in maintaining overall health [56].

The vitamin C (ascorbic acid) content in green onions is very high [57] and plays many important roles in boosting the body’s immune defenses, aiding in collagen production, and having antioxidant properties that protect cells from damage [58]. A study by [59] indicates that a delay in harvesting green onions may cause a substantial yield increment but a concurrent reduction in the content of important nutrient elements such as vitamin C, carotenoids, chlorophyll-a, chlorophyll b, sugars, volatile oils, nitrates and total N, K and Ca. Vitamin K, a vital fat-soluble nutrient, acts as a coenzyme in the carboxylation of specific amino acids, transforming glutamic acid into γ-carboxyglutamic acid [60]. This conversion is crucial for the activation of proteins involved in blood coagulation, making vitamin K essential for the body’s ability to form blood clots and control bleeding [61]. Rich dietary sources of vitamin K include green leafy vegetables like green onions as well as certain plant oils such as canola oil and soybean oil; ensuring an adequate intake of this nutrient can support proper blood clotting and overall health [62]. Vitamin K in green onions is involved in blood clotting and bone health [63].

### 5.2. Dietary Fiber

Dietary fiber constitutes a diverse combination of compounds, primarily consisting of non-starch polysaccharides like cellulose, hemicelluloses, pectins and lignin, along with substances such as gums, resistant dextrins and resistant starches, all of which resist digestion in the small intestine [64]. These indigestible components of fiber provide numerous health benefits, including promoting digestive regularity, reducing cholesterol levels and helping to control blood sugar, making them an essential part of a healthy diet [65]. Fiber-rich foods have the unique ability to create a sense of satiety and fullness while providing minimal calories, making them a valuable tool in weight management by curbing overeating and promoting weight loss, as well as helping to maintain healthy blood pressure levels [66]. Green onions are a source of dietary fiber (non-digestible carbohydrates), which is important for a healthy digestive system [62]. Fiber aids in maintaining regular bowel movements, preventing constipation by adding bulk to the stool for easier passage and promoting a feeling of fullness, which can be helpful for weight management [62]. A diet rich in fiber supports heart health by lowering cholesterol levels and by regulating blood sugar, making green onions a favorable choice for people who are at risk of developing diabetic and cardiovascular disease conditions [65].

### 5.3. Calories and Fat

Green onions are low in calories (energy intake) and fat content, making the crop one of the most suitable vegetable commodities for maintaining a healthy weight [67]. Typically, green onions contain approximately 32 kcal calories per 100 g of fresh weight [63]. Their low calorie content implies that a substantial amount of green onions may be consumed without significantly impacting the daily caloric intake required by the body. By incorporating more vegetables, such as green onions into one’s diet, individuals can effectively lower their consumption of saturated fats and high-calorie foods. The low calorie content implies that a substantial amount of green onions may be consumed without significantly impacting the daily caloric intake required by the body, potentially contributing to the development of a healthier and more balanced eating pattern [24]. This dietary shift towards vegetables not only promotes better nutrition but also aligns with a broader strategy for overall health by reducing the intake of less nutritious options, thereby supporting a more wholesome and well-rounded diet. This property makes green onions an excellent addition to any meal plan for individuals seeking to control their energy intake and is thus essential for weight management. Generally, green onions are virtually fat-free, containing just about 0.2 g of fat per 100 g [63], which is appreciated for maintaining a healthy weight. When fewer calories are consumed than expended, the body starts to utilize its stored fat for energy, leading to weight loss or weight maintenance. Dietary fat is more calorie-dense than protein and carbohydrates, making the consumption of low-fat vegetables like green onions more convenient.

### 5.4. Minerals

Plants play a crucial role in providing essential minerals necessary for maintaining a healthy human body [2]. A variety of vegetables and fruits serve as abundant sources of major minerals like potassium and calcium, both of which are integral to various physiological functions [48]. Green onions, like many other fresh vegetables, are noted for their rich source of these vital minerals, including potassium, magnesium, phosphorus and calcium, making them a valuable addition to a balanced diet [68]. Potassium is essential for maintaining proper nerve function, muscle contractions and regulating blood pressure. Calcium, on the other hand, is crucial for bone health, blood clotting and muscle function. Both of these minerals are integral to maintaining overall health, and green onions contribute to meeting the body’s requirements for these minerals [48]. Incorporating green onions into one’s diet not only adds flavor and nutrition but also helps ensure an adequate intake of essential minerals, further emphasizing the importance of including a variety of plant-based foods to support human health and well-being. Micronutrients, also known as trace minerals, have enormous health benefits. Manganese in green onions contributes to bone formation, blood clotting and a healthy metabolism in the human body [69]. A nutritional component analysis of green onions based on powder extract reveals that green onions are a source of iron and zinc [20]. In the human body, iron forms complexes with molecular oxygen in hemoglobin and myoglobin [70]. Along with iron, zinc is crucial for bodily functions, such as oxygen transport, immune system support and overall human health. Zinc is an indispensable trace mineral with diverse roles in the body. Structurally, it is an integral component of numerous proteins, contributing to their stability and function.

## 6. Therapeutic Value of Green Onions

**Figure 5:**
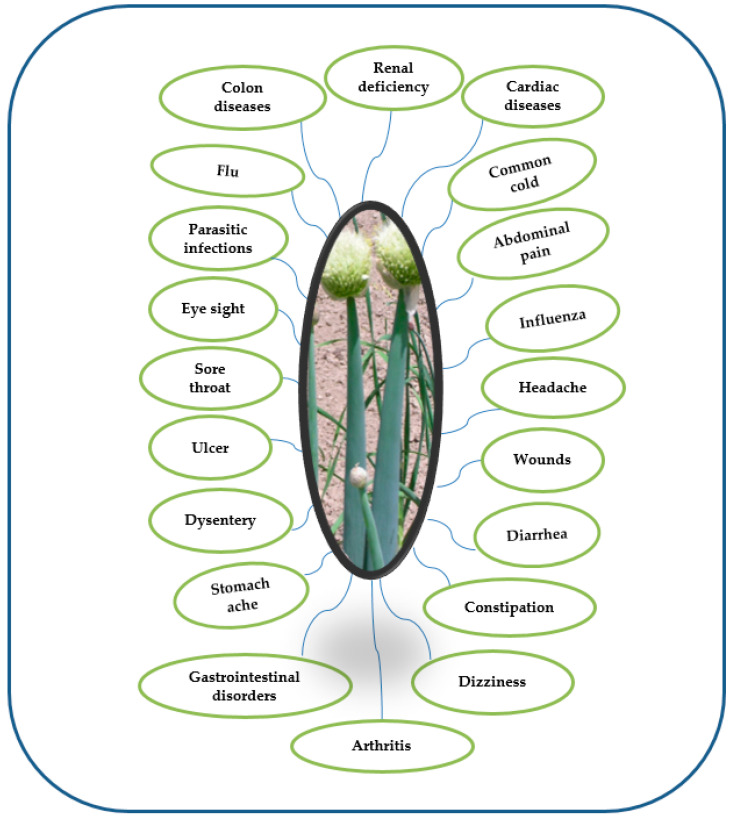
Traditional uses of *Allium fistulosum* as a herbal medicine for treatment of various diseases based on literature reports.

**Figure 6:**
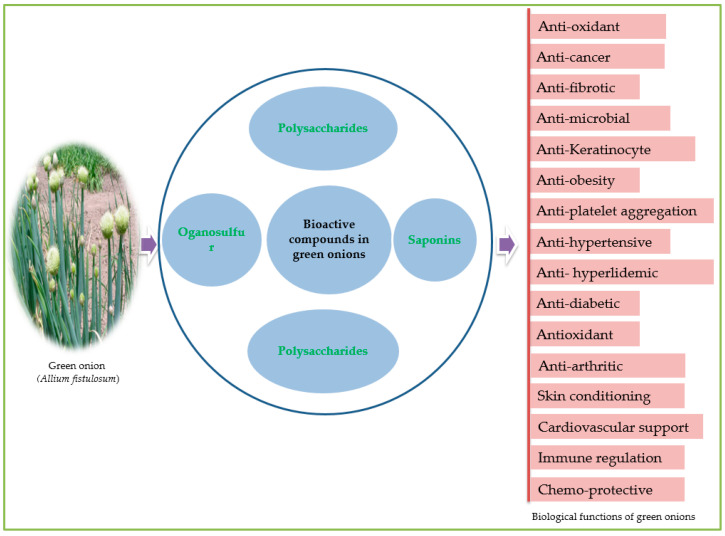
Representative biological functions of green onions.

Bioactive components in vegetable foods or plant compounds serve as therapeutic agents in disease prevention and treatment while optimizing various physiological functions [6]. Biochemically, what distinguishes various vegetables is their unique blend of these chemicals, offering distinct structural characteristics and therapeutic properties. The therapeutic potential of edible Alliums has been acknowledged across cultures and medical traditions [71]. These vegetables have been employed for their diverse biological functions, including antimicrobial, anti-inflammatory and immune-boosting functions, promoting cardio-protective health, digestive health and have anticancer properties [72] (Figure 5). Much of this therapeutic potential can be attributed to the presence of bioactive compounds, particularly organosulfur and phenolic compounds, in *Allium* vegetables [73]. One noteworthy example is *Allium fistulosum*, whose leaves, pseudostem, non-developed bulbs, roots, flowers and seeds are used in medicinal preparations [53]. All the plant parts contain useful phytochemicals that are associated with diverse biological activities [74] (Figure 6). Like other *Allium* species, the green onion is endowed sulfur-containing compounds, such as allicin, alliin and ajoene, as well as flavonoids, for example, quercetin and kaempferol [46]. These compounds are known for their potent antimicrobial properties and have been used traditionally to combat infections [75]. Moreover, the organosulfur compounds in green onions contribute to their anti-inflammatory effects, making them a valuable resource in managing inflammatory conditions [33]. Recent research has shed light on the immune-boosting potential of green onions. For instance, in a study that investigated the effects of green onion leaf extract (GLE) on immune competence in a human clinical trial, Hirayam et al. [76] revealed the potential of an intake of high-or-low amounts of GLE in regulating human adult immune competence and preventing infectious diseases such as common colds and influenza. Compounds like quercetin and vitamin C found in green onions can enhance the immune system’s response to infections and potentially reduce the severity and duration of illnesses [53]. The fiber content in green onions also supports digestive health by aiding in regular bowel movements and maintaining a healthy gut microbiome [7]. Antioxidants, including various flavonoids and phytochemicals in green onions, help protect cells from oxidative stress and may play a role in reducing the risk of chronic diseases [77]. Its phytonutrient-rich composition contributes to its medicinal properties, making it valuable in traditional and modern medicine. Traditionally, extracts of the crop have been used for treating diseases, including common colds, abdominal pains, influenza, headaches, eye sight, wounds, diarrhea, stomach pains, constipation, ulcers, dysentery, parasitic infections, sore throats and several others [14]. The phenolic extract of green onion roots was reported to contain some 31 compounds, including phenolic, flavonoid and tannin [23]. These bioactive compounds (flavonoids and organosulfur compounds) possess anti-inflammatory, antioxidant and antimicrobial properties and, when consumed in diets or used in medicinal preparations, contribute essentially to maintaining a healthy human body [58]. They contribute to disease prevention and management, including cardiovascular diseases and cancer [78]. This vegetable serves as a prime illustration of how plant-based compounds in nutraceuticals can be harnessed to improve human health, showcasing the importance of a natural and holistic approach to healthcare that leverages the healing potential of nature. The biological functions of *A. fistulosum*, including anti-inflammatory, antimicrobial, anti-arthritic, antiobesity, antioxidant and anticancer functions [48], are highlighted further in Section 5.1, Section 5.2, Section 5.3, Section 5.4 and Section 6.

### 6.1. Anti-Inflammatory Activity of Allium fistulosum

Inflammation is a complex and orchestrated host defensive response that the body initiates amid various triggers, such as harm inflicted by infected tissues, pathogenic infections, injurious chemicals and diverse external threats [79]. Inflammation occurs as a response that is manifested through observable symptoms like localized redness, swelling, oedema, warmth, heightened temperature, pain and impaired function of the affected area [80,81]. This biologically reactive mechanism helps in preventing further damages that may develop from the harmful triggers and, consequently, initiates the healing process of already affected tissues [79]. It involves various cells and molecules working together to address the threat [82]. The redness and heat result from increased blood flow to the affected area. Swelling occurs due to the accumulation of fluid and immune cells. Pain serves as a warning signal, while dysfunction highlights the need for rest and repair. Ultimately, inflammation is a fundamental aspect of the body’s defense and repair mechanisms [83]. There are various plant bioactive compounds with significant roles as anti-inflammatory compounds or agents that play significant roles in easing or healing inflammatory reactions or symptoms in the body [84]. *Allium* vegetables, such as green onions, garlic, onions and leeks, are renowned for their notable roles in combating inflammation within the body [85]. The presence of assorted bioactive compounds in these edible vegetables, including allicin, quercetin and various sulfur-containing compounds, is the reason for their potent anti-inflammatory properties [86].

Green onions, in particular, exhibit remarkable anti-inflammatory activity due to their unique combination of phytonutrients [53] (Figure 6). These compounds not only help reduce inflammation but also bolster the body’s defense against oxidative stress and are a rich source of antioxidants, including vitamin C and flavonoids, which are crucial in quelling inflammatory responses [69]. Anti-inflammatory potential is linked to conditions like arthritis, cardiovascular disease and even some cancers [48]. In a study to determine the anti-inflammatory, analgesic, phytochemical and safety effects of *A. fistulosum* in mice, Nazir et al. [87] found that the ethanol extract of *A. fistulosum* provides a beneficial therapeutic effect that eases pain and inflammation. The anti-inflammatory property of the plant was attributed to the abundance of pharmacologically active compounds, including flavonoids, carbohydrates, amino acids, glycosides, phenols and tannins.

### 6.2. AntiMicrobial Activity of Allium fistulosum

In recent years, there has been a significant increase in awareness regarding the vast number of microorganisms that coexist with humans in our environment, leading to a continuous stream of groundbreaking findings on the crucial roles microorganisms play in influencing our susceptibility to various diseases [88,89]. It is intuitive that microbial organisms have and will continue to pose a profound impact on human well-being. There has also been an accelerated pace in understanding the intricate relationships between our microbiomes (the collection of all microbes, viruses, fungi, bacteria and their genes that naturally inhabit our bodies) and human health and drug discovery, such as exploring the potential applications of the phytochemicals of plant bioactive compounds [90]. *Allium* species, which include garlic, onions and green onions, have a long history of use for their medicinal properties [91]. The range of bioactive compounds, including sulfur-containing compounds and flavonoids, present in these edible crop species have antimicrobial properties against the pathogens of both plants and animals [10]. For instance, the bioactive compounds in green onions exhibit remarkable antimicrobial properties [92] (Figure 6). These compounds can inhibit the growth and activity of various microbial organisms, including fungi, bacteria, viruses and nematodes [93].

Chang and colleagues [8] conducted a comparative study of the total antioxidant and antimicrobial potentials of ethanolic extracts from various organ tissues of Alliums. The study revealed that the stem extract of *A. fistulosum* L. is more potent in inhibiting Bacillus subtilis, a Gram-positive model bacterium frequently used for the study of physiology and metabolism and as an industrial organism for protein secretion [94]. The antimicrobial properties of *A. fistulosum* were demonstrated in a study by Sohn et al. [93]. In this study, fistuloside A, B and C compounds were isolated from *A. fistulosum*, and their antimicrobial activity was tested with microorganisms. Their results indicated that these compounds are strong against fungal microorganisms, which suggests the potential of utilizing *A. fistulosum* in the treatment of microbial infections. The use of extracts from Alliums in the treatment of human respiratory diseases such as common colds and flu can be traced to ancient days. The influenza virus causes flu, an acute respiratory disease in humans. In their study, using mice inoculated with the influenza virus, Lee et al. [95] showed that fructan extracted from the green leafy part of *Allium fistulosum* possesses an active anti-influenza virus property by inhibiting the replication of the virus. This confirms the antimicrobial property of *A. fistulosum*. While these natural antimicrobial agents hold promise, further research is needed to fully elucidate their mechanisms and potential applications. Nonetheless, incorporating *Allium*-rich foods like green onions into our diets can offer a flavorful and natural means of supporting human well-being.

### 6.3. Anti-Arthritic Properties of Green Onions

Arthritis, a medical condition closely linked with inflammation, affects millions of people worldwide [82]. It occurs as the inflammation of one or more joints in the body, leading to pain, joint dysfunction, bone damage, swelling and decreased mobility [96]. Two of the most common types of arthritis are osteoarthritis and rheumatoid arthritis, both of which can cause considerable discomfort and impairment in our daily lives [97,98]. Osteoarthritis is characterized by the wear and tear of joint cartilage over time, often seen in aging individuals or those with joint injuries. Rheumatoid arthritis, on the other hand, is an autoimmune disorder where the immune system mistakenly attacks the synovium, the lining of the membranes that surround the joints [97]. Regardless of the type, the common denominator in arthritis is inflammation, which plays a pivotal role in the progression of the disease. The role of plant bioactive compounds as anti-arthritic agents has been investigated [96]. In recent years, there has been earnest interest in identifying plant species endowed with biochemical compounds for use along with synthetic drugs in the medical treatment of various human diseases [99]. Bioactive compounds, including allicin, quercetin and other sulfur-containing compounds, in green onions have gained recognition for their potential anti-arthritic properties, alleviating arthritis symptoms [77]. By reducing inflammation in the affected joints, these bioactive compounds help mitigate the pain and swelling that is experienced [33]. Moreover, the antioxidant properties of green onions’ compounds aid in neutralizing harmful free radicals that contribute to the joint damage seen in arthritis [92]. This dual action of reducing inflammation and combating oxidative stress makes green onions a valuable dietary addition which helps relieve arthritis symptoms. In a study that examined the effects of aqueous extracts of *A. fistulosom* on bone growth based on the calcium- and vitamin D-deficient model, Ryuk et al. [77] revealed the potency of *A. fistulosum* extracts in facilitating bone growth in children and adolescents via increasing the growth plate with no adverse side effects. No metabolic disorders nor release of obesity-inducing hormones were observed.

### 6.4. Antiobesity Properties of Green Onions

Obesity is a complex and multifaceted health condition and a global public health concern [100]. It is characterized by the excessive accumulation of adipose tissue or fat in the body and is primarily caused by an imbalance between the number of calories consumed and the amount of energy expended. This imbalance leads to a surplus of energy in the form of calories, which the body stores as fat. Over time, this excess fat accumulation can result in a range of adverse health effects. The fundamental cause of obesity is an overconsumption of calories relative to the calories burned through physical activity and metabolic processes. This overconsumption can be attributed to various factors, including dietary choices and eating habits [67]. High-calorie, low-nutrient foods, often referred to as “empty calories”, are a significant contributor to this problem. These foods are typically rich in sugars, fats and processed carbohydrates, which are not only energy-dense but also lack essential nutrients. Additionally, the prevalence of fast food and convenience meals has made it easier for individuals to access calorie-dense, unhealthy options, contributing to an overall imbalance in caloric intake [101]. Conversely, the amount of energy expended is influenced by physical activity, the metabolic rate and genetic factors. Sedentary lifestyles, characterized by prolonged periods of sitting and minimal physical exertion, have become increasingly common in modern society. This lack of physical activity means that fewer calories are burned, exacerbating the caloric imbalance that underlies obesity. Moreover, genetic factors can play a role in an individual’s propensity to gain weight, as they influence factors such as metabolism and fat storage. Obesity is a critical risk factor responsible for a wide array of chronic diseases, including diabetes, cancer, hypertension, atherosclerosis and cardiovascular disease [7]. The adverse effects of obesity on health also extend to an increased risk of sleep apnea, joint problems and mental health issues such as depression and anxiety. Moreover, the inflammatory state induced by excess fat can contribute to a chronic low-level inflammation in the body, further promoting the development of chronic diseases.

Edible *Allium* vegetables, such as green onions, have gained attention for their potential role in promoting weight management and thus serving as an antiobesity agent [7]. Green onions’ bioactive compounds, including flavonoids, organosulfur compounds and other phytochemicals, are believed to play a role as antiobesity agents in various ways, such as through appetite regulation to potentially reduce food intake, the enhancement of thermogenesis, burning calories to produce heat, preventing excess fat storage, and consequently, contributing to weight management [102]. In their study that investigated the effects of *A. fistulosum* extracts on body weight and obesity-related conditions, Sung et al. [7] revealed the significant role of A fistulosum extract in weight management and attenuating high-fat-diet-induced obesity. Previously, Sung and co-workers [102] investigated the antiobesity activity and underlying mechanism of a 70% ethanol extract from *Allium fistulosum* in high-fat-diet-induced obese mice. Their findings revealed that the extract did not only significantly reduce the body weight and white adipose tissue (subcutaneous, epididymal and retroperitoneal) weight of the mice, but the adipocyte size compared to high-fat-diet-induced control mice was also decreased. This finding also supports the potential role of *Allium fistulosum* in obesity management. In another study, Sung and colleagues [67] investigated the nutritional composition and antiobesity activities of cereal bars containing *Allium fistulosum* extract using high-fat-diet-induced obese mice. Their results indicated that extracts from *Allium fistulosum* were not only rich in vitamins C, B_2_, B_3_ and B_9_ and protein but also reduced body weight, lipid accumulation in the liver and adipose tissue, as well as adipocyte size in the obese mice. The results indicate the prospects of *Allium fistulosum* as an essential nutraceutical for the management of obesity and metabolic disorders [67].

Thus, the consumption of diets containing aromatic vegetables as well as nutraceuticals from the crop helps in the treatment of obesity (Figure 6) and its associated illnesses, including diabetes, hypertension (high blood pressure) and cardiovascular diseases.

### 6.5. AntiOxidant Properties of Green Onions

Reactive Oxygen Species (ROS) are chemically reactive molecules that are generated in response to oxidative stress [103]. ROS are generated as natural by-products of oxygen metabolism during various cellular metabolism processes [104]. The biosynthesis of ROS primarily occurs through the electron transport chain (ETC) in mitochondria during oxidative phosphorylation [105]. Thus, mitochondria, the cellular powerhouses and other cellular compartments, including the endoplasmic reticulum and peroxisomes, are the primary sources of ROS production [106]. ROS encompass a range of molecules, including superoxide anions (O_2_^•−^), hydrogen peroxide (H_2_O_2_), hydroxyl radicals (·OH), the neutral form of the hydroxide ion (OH ^−^) and singlet oxygen (O_2_). The superoxide anion is a primary ROS generated in biological systems and serves as a precursor for other, more potent forms of ROS, such as hydrogen peroxide [107]. ROS have a Janus-faced nature within the human body. In one form, in moderate amounts, ROS function as signaling molecules involved in various physiological processes, including immune response, cell proliferation and apoptosis [108]. They also participate in the body’s defense mechanisms against invading pathogens [109]. However, when ROS levels are extreme, oxidative stress then occurs [108]. Overwhelming levels of oxidative stress can disrupt the body’s antioxidant defense systems, consequently resulting in damaged cell membranes and various essential molecules such as lipids, proteins and DNA [110]. This condition further leads to uncontrolled cell division, inflammation, cellular dysfunction and a plethora of pathogenic conditions, thus predisposing the body to various diseases, including cancer, neurodegenerative disorders, arthritis, inflammatory disorders, diabetes, hypertension, arthrosclerosis, cardiovascular diseases, aging and many more [111]. Antioxidants are compounds that counteract the harmful effects of oxidative stress by neutralizing ROS and free radicals [112]. They are essential for the body’s defense against oxidative damage, which is implicated in various diseases, including cancer, cardiovascular diseases and neurodegenerative disorders [113]. Common antioxidants include vitamins (e.g., vitamin C and vitamin E), minerals (e.g., selenium) and various phytochemicals found in fruits, vegetables and herbs [114].

The edible members of the *Allium* genus, including green onions (*Allium fistulosum*), garlic (*Allium sativum*) and onions (*Allium cepa*), are renowned for their rich content of bioactive compounds with significant antioxidant properties [115]. Green onions are a rich source of flavonoids (quercetin and kaempferol), sulfur-containing compounds (mainly allicin), vitamin C, minerals and phenolic compounds, such as ferulic acid, which are important antioxidant compounds (Figure 6), protecting cells from oxidative damage [92]. In their study, Wang et al. [116] revealed that extracts from all parts of the plant, the pseudostem [75], leaf and root, have considerable antioxidant effects, with extracts from the stem showing the highest antioxidant properties [117]. In a study that investigated the antioxidant potential of diverse Alliums, Stajner et al. [57] found that all *Allium* species contained high concentrations of bioactive compounds, including flavonoids and carotenoids, as well as low concentrations of toxic oxygen radicals with strong antioxidative activities. *Allium fistulosum* L. was described as the most powerful naturally occurring non-toxic antioxidant, ideal for use in the food, pharmaceutical and cosmetic industries [57]. Yamamoto and colleagues [118] studied the effects of Welsh onion on the development of hypertension and autoxidation in rats. Their results revealed that the green, leafy Welsh onion reduced superoxide generation by suppressing angiotensine II production and NADH/NADPH oxidase activity and lowered the blood pressure in rats fed with a high-fat–high-sucrose diet. This reveals the potential of *A. fistulosum* in attenuating oxidative stress.

In another study, the antioxidative property of aqueous extracts from green leaves of *A. fistulosum* was examined, with findings that the extracts reduced excessive lipopolysaccharide (LPS)-induced nitric oxide (NO) generation and inhibited the expression of Cyclooxygenase 2 (COX-2), an enzyme involved in the production of protagladins during inflammation (Borsen Wang and co-workers, 2005). The potential biological functions of *A fistulosum* extracts in promoting anti-inflammation, preventing atherosclerosis and protecting patients with cardiovascular lesions were also highlighted [119].

### 6.6. AntiCancer (AntiTumor) Properties of Green Onions

Cancer is a complex group of diseases that can occur in virtually any tissue or organ and can affect people of all ages [120]. The disease is among the leading causes of human death worldwide, thus posing a considerable global health burden [121,122]. In 2020 alone, an estimate of human deaths across 185 countries that were associated with varying forms of cancer was 19.3 million [121]. Regardless of the challenges resulting from this condition, persistence in medical research continues to improve our knowledge and the treatment options that can be applied. Plant bioactive compounds have gained considerable attention for their potential anticancer properties [123]. These compounds, often found in fruits, vegetables and herbs, can play a crucial role in preventing and inhibiting the growth of cancer cells.

Green onions and other edible *Allium* species are promising anticancer agents, attributable to their rich content of bioactive compounds [124]. The crop, particularly abundant in organosulfur compounds, flavonoids and other phytochemicals, is of great promise as a cancer therapy [33] (Figure 6). Organosulfur compounds, such as allicin, are known for their ability to induce apoptosis (programmed cell death) in cancer cells and inhibit their proliferation, while flavonoids possess antioxidant properties that help neutralize harmful free radicals and reduce oxidative stress, which can lead to cancer [125]. Research has shown that green onions may have a role in preventing various types of cancer, including colorectal, gastric and lung cancer [125]. They can potentially interfere with the development and progression of cancer by blocking key pathways and promoting the destruction of malignant cells. The potential anticancer effects of green onions are part of a broader trend in studying plant-based diets and their impact on cancer prevention. In a study aimed at investigating the in vitro effect of *A. sativum* and *A. fistulosum* as therapeutic agents, Tigu et al. [33] found that at a high concentration of application of extracts from these Alliums, human fibroblasts and human keratinocyte growth were inhibited. This finding indicates the role of *A. fistulosum* and other Alliums in the treatment of cancer conditions. The chemoprotective effects of extracts from *Allium fistulosum* on colon cancer were invested in a study using a mouse model of colon carcinoma (CT-26 cells subcutaneously inoculated into BALB/c mice) [126]. The results of the study revealed that the extracts inhibited certain key markers associated with inflammatory conditions such as COX-2 and iNOS. Also, the extract suppressed the expression of several cellular markers linked to tumor apoptosis, proliferation, angiogenesis and tumor invasion in mice, indicating the potential role of the dietary intake of *A. fistulosum* in lowering the risk of colon cancer

## 7. Conclusions and Future Perspectives

Green onions have proven to be an essential aromatic vegetable crop with immense value in terms of food, nutrition and therapeutic significance. From a nutritional perspective, green onions are a rich source of vitamins, minerals and antioxidants. They provide vital nutrients, such as vitamins, minerals and several nutrient compositions, which play crucial roles in maintaining overall human health. The presence of flavonoids and organosulfur compounds in the crop offers protection against various chronic diseases. Extracts from green onions have been used for their potential properties, including as agents of anti-inflammatory, antimicrobial, anti-arthritic and anticancer conditions. The therapeutic significance of green onions, particularly in traditional medicine, has been recognized for centuries. Looking ahead, there are several exciting prospects for green onions in terms of their applications, the industrial extraction and processing of useful compounds and research advancement. Due to the ever-growing interest in sustainable agriculture and a shift toward plant-based diets, green onions are poised to play an even more significant role in our culinary and nutritional landscapes. Industrial extraction provides opportunities to isolate and process these essential compounds for their applications in pharmaceuticals, functional foods and natural preservatives. Continued research advancements in various areas such as genomics, metabolomics and biotechnology will help unveil further complex mechanisms in the compounds for their properties and potential applications in agriculture, health and sustainable resource utilization. The cultivation of green onions can be optimized through innovative and environmentally friendly agricultural practices, reducing water and pesticide use and adopting regenerative farming techniques to make production more sustainable and ecologically responsible.

## Data Availability

Not applicable.
